# Scombrotoxinism: Protracted Illness following Misdiagnosis in the Emergency Department

**DOI:** 10.1155/2015/597934

**Published:** 2015-08-19

**Authors:** Ghan-Shyam Lohiya, Sapna Lohiya, Sunita Lohiya, Vijay Krishna

**Affiliations:** ^1^Occupational Medicine & Toxicology, Royal Medical Group, 1120 W. Warner Avenue, Santa Ana, CA 92707, USA; ^2^University of Washington, Seattle, WA 98104, USA; ^3^Royal Medical Group, 1120 W. Warner Avenue, Santa Ana, CA 92707, USA; ^4^MidMichigan Medical Center, Midland, MI 48640, USA

## Abstract

*Background*. Scombrotoxinism is an acute toxin-induced illness caused primarily by bacterial synthesis of histamine in decomposed fish. *Case Report*. Immediately after taking 2-3 bites of cooked salmon, a clerical worker developed oral burning, urticaria, and asthma. In the emergency department, she was diagnosed with “allergies”; scombrotoxinism was never considered. She then developed wide-ranging symptoms (e.g., chronic fatigue, asthma, anxiety, multiple chemical sensitivity, and paresthesiae) and saw many specialists (in pulmonology, otorhinolaryngology, allergy, toxicology, neurology, psychology, and immunology). During the next 500+ days, she had extensive testing (allergy screens, brain MRI, electroencephalogram, electromyogram, nerve conduction velocity, heavy metal screen, and blood chemistry) with essentially normal results. She filed a workers' compensation claim since this injury occurred following a business meal. She was evaluated by a Qualified Medical Evaluator (GL) on day 504, who diagnosed scombrotoxinism. *Comment*. Scombrotoxinism should be considered in all patients presenting to the emergency department with “oral burning” or allergy symptoms following “fish consumption.” Initial attention to such history would have led to a correct diagnosis and averted this patient's extended illness. Specialist referrals and tests should be ordered only if clinically indicated and not for diagnostic fishing expedition. Meticulous history is crucial in resolving clinical dilemmas.

## 1. Introduction

Scombrotoxinism is an acute self-limiting but potentially serious illness produced by consuming fish containing scombrotoxin, a mixture of histamine and related amines [1–5]. Under suboptimal refrigeration, contaminating anaerobic bacteria thrive in fish, and their carboxylase enzyme converts fish histidine into histamine. Ingestion produces immediate effects of histamine* toxicity* mimicking type-1 allergy. The affected fish need not look spoiled or malodorous. Scombrotoxin is heat-stable and therefore is not inactivated by cooking. Fish histamine concentration >50 ppm suggests decomposition. Scombrotoxinism responds well to antihistamines and corticosteroids [1–5]. We report a case of occupational scombrotoxinism where its correct diagnosis eluded recognition and led to a complex illness involving extensive medical care and disability. Real life lessons from this workers' compensation claim may help improve future case management.

## 2. Case Report

Immediately after taking 2-3 bites of cedar-plank-salmon during a business dinner, a clerk experienced “peppery burning in mouth, choking sensation, facial flushing, chest tightness, dizziness, and palpitations” (day 1). She had previously consumed salmon uneventfully many times. As first aid, she took 50 mg of oral diphenhydramine. Her weight was 93 Kg and height 172 cm; body mass index was 31 Kg/SqM (obese). She then received IV corticosteroids in an emergency room, followed by oral prednisone (50 mg daily, tapered 10 mg weekly to 10 mg daily maintenance dose). The diagnosis was an unspecified “allergic reaction.” Although required, no report was made to the Health Department for this “foodborne and unusual” illness [6]. No toxicology or microbiologic test was performed on the fish.

Patient's symptoms persisted. On day 3, she was hospitalized and treated with IV corticosteroids, ranitidine, and cetirizine. She had moderate microcytic hypochromic anemia (hematocrit: 31.8%, normal 36–47%) probably from chronic menorrhagia. On day 4, she again required emergency room treatment.

For the next 4 months, patient experienced extreme fatigue, mostly remained in bed and saw several physicians. An otorhinolaryngologist diagnosed new asthma and prescribed beclomethasone and albuterol inhalers and allergen avoidance (HEPA air purifier, bedding covers). From day 28 onwards, a pulmonologist saw her monthly, recorded normal clinical examination and spirometry but continued the bronchodilators. A therapist diagnosed severe anxiety disorder from the allergic reaction, documented multiple symptoms (anxiety, excessive wariness, obsessions, compulsions, sleeplessness, and anticipatory fear of another reaction causing death or severe disability), and provided 40+ weekly psychotherapy treatments.

On day 191, a neurologist ordered five tests for patient's dizziness and paresthesiae. Brain MRI showed one hyperintense lesion perpendicular to the ventricular system (an unknown bright object) representing a nonspecific finding, lacunar infarct, or demyelinating disease. Electroencephalogram revealed intermittent nonspecific focal transient theta waves over the temporal lobes. Electromyogram and nerve conduction studies in both lower extremities were normal. Auditory evoked response test revealed normal hearing levels and brainstem activity. On day 219, the neurologist wrote “brain MRI and EEG changes are consequence rather than the cause of her problem,” and prescribed gabapentin 200 mg daily. Repeat brain MRI 5 months later remained unchanged. Yet the neurologist recommended another MRI and cerebrospinal fluid testing.

Patient began developing dizziness and chest tightness following exposure to certain hair dyes, foods (potato chips), iron supplements, toiletries and cleaning chemicals. Monosodium glutamate sensitivity was suspected. The neurologist now advised hair analysis for “any stored neurotoxins.” He also suspected “immune dysfunction.” An immunologist found negative extractable-nuclear autoantibodies, negative antinuclear antibody, normal erythrocyte sedimentation rate, and normal serum thyroid stimulating hormone and immunoglobulins A, E, G, and M.

Since patient's illness occurred during the course of a business meal, she was eligible for workers' compensation benefits. To determine compensability (causation), patient was evaluated on days 133 and 193 by a defense allergist who diagnosed hypertension, anxiety, and nonoccupational allergies. He reported normal results for plasma norepinephrine, dopamine, vanillylmandelic acid (for pheochromocytoma), urine 5-hydroxyindoleacetic acid (for carcinoid tumor), serum tryptase (for mastocytosis), immunoglobulin E, serum chemistry panel, and blood mercury (patient: <3 mcg/L, acceptable: <6 mcg/L). Serum allergy testing for 49 allergens and salmon revealed only dust mite allergy. Patient refused skin allergy testing.

A toxicology evaluator found normal levels of blood lead, arsenic, mercury, and cadmium. Repeat testing for 21 respiratory and ten food allergens was mildly positive for only dust mites.* Helicobacter pylori* antibody was absent. He diagnosed Multiple Chemical Sensitivity and phobia from the severe salmon-related reaction.

To help resolve medical disputes about causation, treatment, and permanent disability, patient was evaluated on day 504 by author GL for an independent State Panel Qualified Medical Evaluation (Occupational Medicine & Toxicology). He diagnosed scombrotoxinism based on a meticulous history and review of records.

## 3. Discussion

This was a classic case of scombrotoxinism. Patient developed oral burning, facial flushing, angioedema, and asthma immediately after salmon consumption. Although the diagnosis was never confirmed by determining the salmon's histamine content,* such incompleteness was inconsequential*. Extensive testing had ruled out all other competing diagnoses. In clinical practice, one may not achieve the level of diagnostic purity sought in research settings. Yet, this real life case offers unique and educationally useful insights to emergency physicians, toxicologists, and other specialists.

This case highlights the frequent underdiagnosis of scombrotoxinism. Despite this patient's tell-tale presentation, her physicians diagnosed “allergies.” This probably occurred because allergies are common and scombrotoxinism is now rarer due to improved food handling. The emergency treatment of the patient was appropriate because symptoms in both allergies (type-1 hypersensitivity illness) and scombrotoxinism (toxic illness) are caused by histamine [4]. However in allergies the histamine source is* intrinsic (from mast cells and basophils)*, whereas in scombrotoxinism it is* extrinsic* (from decomposed fish). The diagnostic clue for scombrotoxinism is the “histamine-induced oral burning” and the history of fish consumption. The initial misdiagnosis occurred because the emergency physician did not recognize this link.

Scombrotoxin is synthesized by contaminating bacteria at room temperature. Therefore, fish, like any other food, should be handled carefully to avoid contamination and not be left in the “temperature danger zone” (21–40°C) for more than two hours. In this context, it is useful to remember the mnemonic “FAT-TOM” emphasizing the six pillars of food safety that need attention: Food, Acidity, Time, Temperature, Oxygen, Moisture [6]. Prompt notification of the local Health Department is essential to avert outbreaks from the same contaminated fish [7].

Patient had no toxicological permanent disability from this injury. Histamine has a very short half-life in extracellular fluid [8]. As a toxicological principle, a xenobiotic is deemed eliminated from blood after seven half-lives (<1% remainder). Thus patient's histamine-related symptoms ought to have resolved quickly, definitely within a week.

Missed diagnosis had significant adverse consequences in this case. This patient developed anxiety and required extended psychological treatment. She saw many specialists who screened her with numerous tests and diagnosed various unrelated conditions. Had patient learned at the outset that her illness was due to food poisoning caused by* that* particular salmon dish, she might not have developed this extended illness. Patient's ongoing symptoms probably represent an anxiety-panic disorder which requires a psychiatrist.

Illnesses from business meals are usually considered as “arising out of employment,” and accepted for workers' compensation. California's Labor Code section 4600(a) specifies “Medical…treatment that is reasonably required to cure or relieve the injured worker from the effects of his or her injury shall be provided by the employer.” Once a worker files a Workers' Compensation Claim, the employer must pay up to $10,000 for its treatment until the claim acceptance or denial [9]. Since patient's injury arose out of employment (the salmon consumption), the initial emergency treatment was the employer's responsibility. However, law does not require employers to diagnose and exclude all nonoccupational conditions that fall within the differential diagnosis. Patient's extensive testing and care after claim denial was unrelated to the injury.

Concern about mercury poisoning was unjustified. First, fish high on the food chain (swordfish, shark, king mackerel, and tilefish) may contain methyl mercury, but salmon is a low mercury fish (<0.09 ppm mercury) [10]. Second, there could not be sufficient mercury in 2-3 bites of salmon to produce mercurialism. Third, there would be a lag between mercury ingestion and symptom development. Finally, mercury toxicity does not produce immediate angioneurotic edema or asthma. Likewise, there was no indication for hair chemical analysis. Presence of a xenobiotic in hair may indicate exposure (both internal and external) but can neither indicate its source nor link it to an illness [11].

Ciguatera-toxinism was a competing diagnosis because of its fish origin and heat resistance. However it was unlikely because ciguatera toxin is tasteless, involves larger fish, has an 8-hour lag period, and produces vomiting and diarrhea [12]. Staphylococcal food poisoning was unlikely because patient did not experience vomiting, stomach cramps and diarrhea [13]. Assuming that the fish was adequately cooked, all potential fish-borne parasites (*Clonorchis sinensis*,* Anisakis*, and* Diphyllobothrium*) and bacteria (*Salmonella* and* Shigella*) would have been inactivated. Moreover, such infestations and infections have different specific clinical presentations, require an incubation period, and do not produce urticaria.

This case illustrates the need for physicians to base their opinions and recommendations on “evidence and science that is nationally recognized and peer reviewed” [14]. This patient was subjected to many tests without adequate justification. For example, the neurologist only cursorily noted “numbness” (without dermatomal or objective anomalies); yet he ordered nerve conduction velocity and electromyogram. Although the brain MRI revealed only a nonspecific change or mild leukoaraiosis which remained unchanged 5-months later, the neurologist still recommended repeat MRI and lumbar puncture, and then hair analysis and an immunologist. Patient was a nonsmoking office worker with no industrial chemical exposure; yet heavy metal levels were checked repeatedly. Likewise, there was no indication to test for environmental allergies, pheochromocytoma, mastocytosis, or carcinoid syndrome. Consequences of unnecessary testing for healthy patients are retesting, repeat venipuncture, unwarranted anxiety, waste, confusion, and false hope [15].

## References

[1] Murray C. K., Hobbs G., Gilbert G. R. (1982). Scombrotoxin and scombrotoxin-like poisoning from canned fish. *Journal of Hygiene*.

[2] Ward D. I. (2011). ‘Mass allergy’: acute scombroid poisoning in a deployed Australian Defence Force health facility. *Emergency Medicine Australasia*.

[3] Hungerford J. M. (2010). Scombroid poisoning: a review. *Toxicon*.

[4] Numbere N. K., Featherstone P., Cooper H. L. (2010). Scombrotoxin poisoning: an important differential diagnosis for anaphylaxis. *Acute Medicine*.

[5] Centers for Disease Control and Prevention (CDC) (2007). Scombroid fish poisoning associated with tuna steaks—Louisiana and Tennessee, 2006. *Morbidity and Mortality Weekly Report*.

[6] Iowa State University Food safety Lesson. http://www.extension.iastate.edu/foodsafety/Lesson/L4/L4p1.html.

[7] Illinois Department of Public Health http://www.idph.state.il.us/health/infect/ReportDiseasePoster.pdf.

[8] Stone K. D., Prussin C., Metcalfe D. D. (2010). IgE, mast cells, basophils, and eosinophils. *Journal of Allergy and Clinical Immunology*.

[9] http://law.onecle.com/california/labor/4600.html.

[10] Natural Resources Defense Council Consumer Guide to Mercury in Fish. http://www.nrdc.org/health/effects/mercury/guide.asp.

[11] Centers for Disease Control and Prevention (CDC) Hair Analysis. Exploring the state of the science. http://www.atsdr.cdc.gov/HAC/hair_analysis/hairanalysis.pdf.

[12] Davis C. Ciguatera Fish Poisoning. http://www.emedicinehealth.com/wilderness_ciguatera_toxin/article_em.htm.

[13] Centers for Disease Control and Prevention (CDC) http://www.cdc.gov/nczved/divisions/dfbmd/diseases/staphylococcal/.

[14] Lohiya G.-S., Lohiya P., Krishna V., Lohiya S. (2013). Death related to ibuprofen, valdecoxib, and medical errors: case report and medicolegal issues. *Journal of Occupational and Environmental Medicine*.

[15] Lohiya G.-S., Tan-Figueroa L., Lohiya S. (2007). Bloodborne pathogens exposure from occupational fingernail scratches. *Journal of the National Medical Association*.

